# Validation study of ultrasonography versus computed tomography for measuring muscle mass loss in critically ill patients: CT mUS study

**DOI:** 10.1186/s13054-023-04596-2

**Published:** 2023-08-08

**Authors:** Leandro Moreira Peres, Fabio Luis-Silva, Mayra Gonçalves Menegueti, Wilson José Lovato, Douglas Alexandre do Espirito Santo, Mariana Derminio Donadel, Lucas Sato, Carolina Hunger Malek-Zadeh, Anibal Basile-Filho, Olindo Assis Martins-Filho, Maria Auxiliadora-Martins

**Affiliations:** 1 Division of Intensive Care Medicine, Department of Surgery and Anatomy, Ribeirão Preto Medical School, Hospital das Clínicas da Faculdade de Medicina de Ribeirão Preto, University of São Paulo, Av. Bandeirantes, S/N, Ribeirão Preto, CEP 14048900 Brazil; 2 Ribeirão Preto Nursing School, University of São Paulo, Ribeirão Preto, Brazil; 3 René Rachou Institute, Oswaldo Cruz Foundation, FIOCRUZ-Minas, Belo Horizonte, Minas Gerais Brazil

*Trial registration*: This clinical trial is registered at REBEC https://ensaiosclinicos.gov.br/search/query/simple?q=RBR2bzspnz.#gsc.tab=0&gsc.q=RBR-2bzspnz.&gsc.page=1. The protocol was approved, on July 30, 2019, by the Research Ethics Committee of the Hospital das Clínicas, Faculdade de Medicina de Ribeirão Preto—Trial Registration Number: 3,475,851.

Nutritional therapy is important for critical patient care and crucial for recovering from serious illnesses, morbidity and mortality reduction by maintaining the functional integrity of the gastrointestinal tract, decreasing the catabolic response, in addition to contributing to the reduction in length of hospital stay resulting in a lower cost of treatment [1]. Critical patients suffer early changes in the quantity and quality of muscle mass [2]. Tools for identifying the groups most susceptible to these complications are needed so that interventions can minimize the deleterious effects of malnutrition in critically ill patients. This study aimed to compare the thickness of the quadriceps muscle using ultrasonography and computed tomography (CT) in critically ill patients with traumatic brain injury (TBI). This is a prospective validation study with a convenience sample carried out in an intensive care unit (ICU) of a tertiary teaching hospital.

Serial ultrasounds (US) and CT were obtained upon patient admission to the hospital. The second measurement was 24 to 96 h after admission and the third measurement 72 to 168 h after admission. All US measurements were taken simultaneously with quadriceps CT [3]. We have performed 21 measurements to assess the inter-observer correlation in performing the US and found a strong positive correlation (*r* = 0.99) with good limits of agreement using the Bland and Altman analysis. The bias was 0.06 mm with the limit of agreement ranging from − 0.34 to 0.46. We performed 20 measurements to assess the intra-observer correlation, and we found a strong positive correlation (*r* = 0.99 for both observers) with good agreement according to the Bland and Altman analysis. The bias was − 0.06 mm with a limit of agreement ranging from − 0.55 to 0.43 and a bias of 0.06 mm with a limit of agreement ranging from − 0.37 to 0.49, respectively. Fifty patients were eligible for the study. We analyzed 252 images in 49 patients, since 49 participated in the study in time 1 of the images, 40 patients in time 2 and 37 in time 3.

In this study, the reference standard for assessing muscle mass was performed with CT. The accuracy of the assessment of muscle mass by CT is well established, as it is a method that allows separating muscle, fat and other tissues.

The evaluation of the thickness quadriceps muscle between US and CT was evaluated in three measurement times. Spearman's correlation coefficient was used to investigate relationships between measured variables. The coefficients obtained were *r* = 0.95, *p* < 0.01, at time 1 (Fig. 1A), *r* = 0.92, *p* < 0.01, at time 2 (Fig. 1B), and *r* = 0.88, *p* < 0.01, at time 3 (Fig. 1C). In addition to a positive correlation, we observed a high agreement between the methods. The Bland and Altman analysis at time 1 showed the bias of 1.5 with limits of agreement varied between − 3.7 and 6.7 (Fig. 1D). At time 2, the bias was 1.92 with limits of agreement varied between − 5.3 and 9.2 (Fig. 1E). At time 3, the bias of 3.2 mm with limits of agreement varied between − 4.7 and 11.1 (Fig. 1F).

**Fig. 1:**
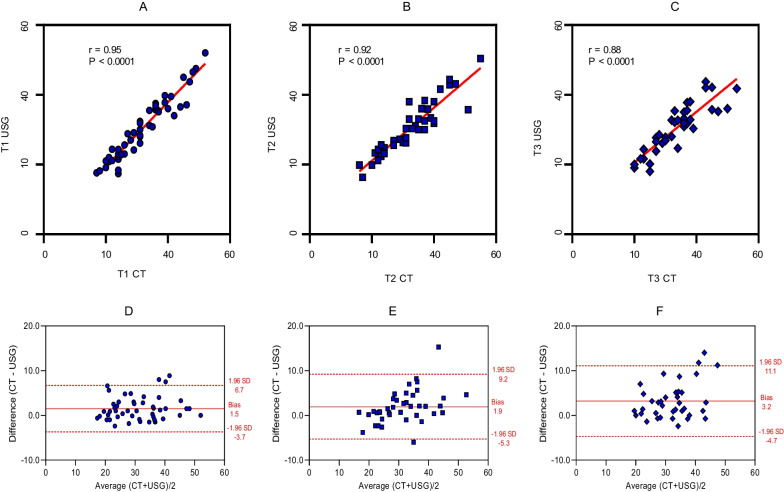
**A** Spearman’s correlation computed tomography (CT) x ultrasound (US) at time 1, **B** Spearman’s correlation CT x US at time 2, **C** Spearman’s correlation CT x US at time 3, **D** Bland and Altman analysis at time 1, **E** Bland and Altman analysis at time 2, **F** Bland and Altman analysis at time 3

The median of the quadriceps muscle thickness (QMT) values was 31 mm for tomography and 30 mm for US at time 1 (*p* = 0.0002), 32 mm for tomography and 30 mm for US at time 2 (*p* = 0.0021) and 34 mm for tomography and 32 mm for US at time 3 (*p* = 0.00002). We observed a strong positive correlation and good concordance of values with small differences between the two methods, which are irrelevant for clinical practice (Fig. 1).

In this study, we proposed the validation of ultrasonography in comparison with the gold standard (CT) to assess the thickness of the quadriceps muscle in critically ill patients who are victims of TBI. The US is a non-invasive, low-cost method, which is performed at the bedside, being feasible in critically ill patients, since the entire arsenal of tools for nutritional assessment is limited when applied to critically ill patients.

Recognition of low musculature on admission to the ICU is important for nutritional intervention and rehabilitation [4]. Low musculature on admission to the ICU poses a risk of mortality and physical disability. Enteral nutrition and early mobilization are recommended in critically ill patients [5]. Most patients showed a decrease in muscle mass, but this was not constant since intramuscular edema is one of the contributing factors. More studies need to be carried out, such as validation protocols comparing US with reference techniques, mainly in critically ill patients with edematous areas, in which muscle mass loss can occur without changes in QMT, where the water content can affect measurements. It is interesting to perform maximum compression protocols by US in addition to echogenicity assessment so that the accuracy of the analysis can be increased. Our results suggest that ultrasonography might be useful as a tool to assess QMT in critically ill patients with TBI, showing good correlation and concordance with CT.

## Data Availability

Data were entered into an online electronic database (Redcap®) and subsequently analyzed using the statistical program *Microsoft Excel®* e *Statistical Product and Service Solutions (SPSS) V.26* (SPSS Inc.®; Chicago, IL, USA). Images were collected directly from the US and CT apparatus and were stored to ensure reliability. All participants received a numerical identification from the study itself. Personal data will be stored in the hospital's digital system, password protected to ensure confidentiality. Any modifications or adverse events related to the protocol were communicated to REBEC.gov and to the Research Ethics Committee and Clinical Research Unit of Hospital das Clínicas da Faculdade de Medicina de Ribeirão Preto by the researcher in charge. All principal investigators have access to the final trial dataset. Datasets used and/or analyzed during the current study are available from the corresponding author upon reasonable request.

## Abbreviations

CT: Computed tomography
ICU: Intensive care unit
QMT: Quadriceps muscle thickness
TBI: Traumatic brain injury
